# Preliminary Study of the Influence of Supplementary Cementitious Materials on the Application of Electro Remediation Processes

**DOI:** 10.3390/ma14206126

**Published:** 2021-10-15

**Authors:** Isabel Martinez, Marta Castellote

**Affiliations:** Eduardo Torroja Institute for Construction Science, Spanish National Research Council (IETcc-CSIC), Serrano Galvache 4, 28033 Madrid, Spain; marta.castellote@csic.es

**Keywords:** concrete, durability, supplementary cementitious materials (SCMs), corrosion, electro remediation, electrochemical techniques

## Abstract

Supplementary cementitious materials (SCMs), based on pozzolanic materials, improve durability against corrosion and mechanical properties of concrete structures by decreasing their permeability. Even though the influence of SCMs on the chloride combination with the cement phases has been widely studied, its effects on electrokinetic remediation processes such as electrochemical chloride extraction (ECE) have not been clarified. For this reason, the influences of two SCMs, fly ash (FA) and blast-furnace slag, on the extraction of chloride through the concrete net pore have been studied in this paper to determine the viability of the application of electrochemical chloride treatments in these structural materials. Alternative electrochemical indicators to the ones included in the standards are also proposed to better determine the final point of the treatment. A cement replacement of 8% on both SCM (FA and slag) has been tested, and in addition to charge density, chloride content, and corrosion measurement at the end of the treatment (included in the standards), different electrical and electrochemical indicators such as electrical resistivity, EIS, or depolarized potentials are used to monitor the ECE. The influence of the treatment on disconnected steel has been also studied. In the case of slag mortar, no steel passivation was reached, while in the case of FA, the passivation of the steels connected to treatment was reached in the same way as in plain CEM I specimens. A degree of protection is also detected in the nonconnected steel, which means that substitution of 8% in FA does not affect treatment efficiency and can also partially protect the metallic elements embedded in the same electrolyte but not connected to the treatment.

## 1. Introduction

The most important problem concerning the expected service life of concrete structures is related to corrosion of the embedded reinforcing steel, which can cause important loss of mechanical and structural properties [1,2,3].

Corrosion of the reinforcing steel is caused by a decrease in the pH of the pore solution in the cementitious matrix due to different aggressive substances that penetrate through the pore structure, interacting with the cement phases. When the pH decreases in the surroundings of the embedded rebar, the passive layer that protects the steel is altered, and corrosion starts. The two main aggressive substances to take into account in this process are the presence of chlorides and carbonation (due to the CO_2_ penetration through the concrete pores). Chlorides contamination in concrete can be due to: the concrete mixture components (contaminated water or aggregates, or additives with high chloride content) or the external environmental exposure.

In this way, the chlorides penetration from aggressive environments is one of the most important causes of corrosion; that is why chloride diffusion through the concrete cover (diffusion coefficient) [4,5] and its combination with the cement phases (combination degree), as well as the critical chloride content that causes steel depassivation, have been highly studied [6,7,8,9,10].

Electro remediation processes can mitigate corrosion, passivating the steel [11,12,13,14]. To remediate chloride attacks, the electrochemical chloride extraction (ECE) method was developed in the 1970s [15], but its application in concrete structures became more popular during the 1990s [16,17,18,19,20,21], when important results were obtained. The purpose of ECE is to mitigate corrosion activity due to the chloride effect, providing long-term corrosion protection of steel reinforcement, re-establishing its self-protection ability due to the increase of pH in the pore solution of the concrete cover. The duration of treatment varies depending on several factors. The most important are the amount of accumulated chloride and its percentage of combination, the permeability of the concrete (total porosity and pore distribution) and the electrical resistivity of concrete, among others. It can take from several weeks up to as much as several months.

Its working procedure is based on the application of a high-voltage DC between the reinforcing steel (acting as a cathode) and an auxiliary external anode to force the migration of chloride ions from the steel to the concrete surface. Different auxiliary anodes have been investigated [22,23,24,25], but the most used is a Titanium (TiMMO) mesh that is placed outside the concrete surface and is in contact with a high conductivity electrolyte solution. Thus, the electro remediation applied to chloride extraction in concrete using impressed current has been used on a relatively large scale with success and important results for its application in concrete structures have been obtained [12,17,26,27], even though some adverse effects have been detected [28,29]. These effects are related to the local increase of the pH due to the hydroxyl and other alkali ions accumulation near de cathode (steel), which can activate potentially reactive aggregates, causing alkali-silica reactions.

The European standard related to the correct application of ECE treatment [30] indicates that the design’s current density must be in the range of 0.5 to 2 A/m^2^ of concrete surface area. During the treatment, a way to monitor its progress is necessary. The criteria concern the content of chloride in contact with reinforcing steel. That means drilling cores, which implies destructive tests. The total chloride content should preferably be less than 0.4% by the mass of cement at the end of the treatment [31,32]. On structures with different environmental exposures in which chloride content is not homogeneous, is difficult to compare the chloride quantification before and after the treatment. In this way, the treatment must be stopped when a decrease of chloride content under the threshold value is determined and a certain accumulative charged density is reached. Electrochemical corrosion parameters such as I_corr_ and E_corr_ are also monitored after the end of the treatment to assure values under corrosion limits.

So, an important gap on the way to monitor the treatment by non-destructive technique was found. In previous works carried out by the authors with an implementation of ECE treatment in chloride-contaminated OPC (Ordinary Portland Cement) mortars [33], other alternative indicators able to be monitored during the treatment using nondestructive techniques were proposed. In present paper, these parameters have been monitored to check its ability to detect the evolution of chloride extraction as well as the influence of the electrolyte resistance on them, using different types of blended cements (adding SCMs).

On the other hand, supplementary cementitious materials (SCMs) contribute to more sustainable construction, reducing the percentage of cement in the concrete. These additions, based on pozzolanic materials, also improve the durability and mechanical properties of concrete structures by decreasing their permeability, which reduces the penetration of aggressive substances, such as chlorides or CO_2_, from the environment. SCMs are recommended by national and international structural concrete regulations (ACI 318, Euro Code 2 or fib model code, among others), especially in aggressive environments. The hydration of blended cement with SCMs is slower than the one of the OCP and depends on the chemical composition, the fineness, and the amounts of reactive phases, that is, the bulk microstructure formed is different and its ability for ion migration also changes. [34,35].

Even though the influence of SCMs on the chloride combination degree in cementitious materials and its ability to migrate through the net pore has been more and more closely studied [36,37,38,39,40], its effects on ECE treatments have not been investigated enough [41,42,43]. In this work, the influences of FA and blast-furnace slag on the ECE treatment have been evaluated to determine side effects or limitation on its application on concrete structures fabricated with these types of blended cements.

## 2. Materials and Methods

### 2.1. Materials

Two prismatic mortar specimens (size 7 × 7 × 7 cm^3^) per mix, with three embedded steel rebars (*S*_a_, *S*_b_, and *S*_c_) were fabricated following the mixture proportions given in Table 1. Five percent chloride by mass of cement was added as NaCl to the mixing water. Before being subjected to an ECE treatment, these specimens were monitored for at least 100 days to quantify the amount of steel corrosion generated by the chloride content.

Six reference specimens from each typology without rebars (size 4 × 4 × 16 cm^3^) were fabricated for the physicochemical, electrochemical, and mechanical characterization. Workability, chemical composition, compressive and flexural strength, porosity and pore size distribution, and electrical resistivity were assessed to quantify the effects of the SCM materials in the mortars.

### 2.2. Steel-Mortar Characterization

The following tests were carried out for the mortars characterization:

- fresh state characterization: workability: slump (EN 12350-2), density (EN 12350-6), and content of occluded air (EN 12350-7);
- chemical characterization by X-ray fluorescence (FRX); sample in pressed powder tablet;
- total porosity and pore size distribution by mercury intrusion porosimetry (MIP);
- pore solution composition by the so-called “pore pressing test,” applying ICP analysis;
- electrical resistivity using the so-called “direct method” (UNE 83988-1:2014);
- compressive and flexural strength (EN 12504-1:2009).

On the other hand, concerning the corrosion of the steel, electrochemical parameters such as electrical resistivity, half-cell potential, corrosion rate, and EIS were evaluated using a device AUTOLAB PGSTAT302 (©Methrom AG) from 24 h until at least 100 days to control not only the corrosion of the rebar before the ECE treatment, but also the behavior against corrosion of the different admixtures and some mortar properties. The experimental procedure followed is the one described in [33].

#### 2.2.1. Corrosion Potential E_corr_

The corrosion potential E_corr_ of the reinforcing steel is measured as its voltage difference against a reference electrode. In this case, Ag/AgCl/3M KCl reference electrode (©Methrom AG) was used. This parameter provides qualitative information about the corrosion risk. The ASTM C876-09 “Standard Test Method for Corrosion Potentials of Uncoated Reinforcing Steel in Concrete,” indicates that fewer negative values than −200 mV (vs. Ag/AgCl/3M KCl) indicate 90% probability of passivation, whereas values lower than −350 mV indicate 90% probability of corrosion. In the range of −200 and −350 mV, it is not possible to determine the corrosion risk using this parameter.

#### 2.2.2. Concrete Resistivity (ρ) and Electrical Resistance (Re)

The concrete resistivity values indicate the water content in the concrete net pore, which is related to the corrosion ability [44]. In the present experimentation, concrete resistivity was calculated in the reference specimens (without embedded rebars) by the so-called “direct method” [44], using alternated current at high frequencies through two external stainless-steel electrodes in two parallel faces of the specimens. Resistivity is calculated using the expression:

(1) $$\rho =R_{e}k_{cell}$$

where *K_cell_* is:

(2) $$K_{cell}=\frac{A}{L}$$

*A*: Electrode Area

*L*: Distance between electrodes

The electrical resistance of the concrete covering was also measured at high frequencies by EIS, with a three-electrode arrangement using an external stainless-steel counter electrode and an Ag/AgCl/3M KCl reference electrode. These measurements were used for the ohmic drop compensation due to the electrolyte resistance in the polarization resistance measurements.

#### 2.2.3. Corrosion Rate, I_corr_

The corrosion rate (I_corr_) or corrosion density provides the amount of metal that has been transformed into oxide, quantifying the corrosion process. In present experimentation, I_corr_ has been calculated using the linear polarization resistance (LPR) or *R*_*p*_ technique [45].

(3) $$R_{p}={\left(\frac{\Delta E}{\Delta i}\right)}_{\Delta E\to 0}$$

The measurement was made by a three-electrode arrangement using the rebar as working electrode, external stainless steel as a counter electrode (CE), and Ag/AgCl/3M KCl as reference electrode (RE). A potential ramp of ±10 mV from the corrosion potential was applied, and the generated current was registered and the ohmic drop was compensated.

The corrosion rate or current density, I_corr,_ is calculated using the following expression:

(4) $$I_{\mathrm{corr}}=\frac{B}{R_{p}}\;$$

where *B*: Tafel constant. According to the bibliography [46,47], the value of the Tafel Constant considered has been 26 mV.

I_corr_ values under the threshold of 0.1–0.2 μA/cm^2^ are considered negligible (passive steel) while values over 1 μA/cm^2^ are considered as high [48], that is, applying the Faraday law, 1 μA/cm^2^ implies a loss of a section of 11 μ/year.

#### 2.2.4. Electrochemical Impedance Spectroscopy (EIS)

This method is based on the application of an alternating signal instead of a direct signal to the rebar acting as a working electrode. In the present experimentation, the three-electrode arrangement was used for the EIS determinations in the specimens with embedded rebars, using the rebar as working electrode (WE), external stainless steel as a counter electrode (CE), and Ag/AgCl/3M KCl) as reference electrode (RE). The two-electrode arrangement was used only to determine resistivity in the reference specimens. In this case, WE+RE are connected and the current is applied from CE to WE+RE.

EIS provides complementary information on the corrosion process, as are the dielectric properties of the electrolyte, or others related to the passive layer on the steel surface, depending on the range of frequencies to be evaluated. About the high-frequency domain, a correlation between the EIS response and the pore solution or structure can be found [49]. As has been mentioned before, the bulk resistivity of the material (ρ) can be also measured using this technique. This parameter joined with the parameters obtained from the high-frequency range, has been used to determine the role of different additions and admixtures in concrete [50].

The low-frequency domain (1–0.01 Hz) determines the steel-concrete system and is where the corrosion mechanism can be studied, as is where the faradaic processes occur.

The effect of cathodic polarization in the EI spectra has been studied in previous researches [51]. Thus, the present study was focused on this range of frequencies. Displacement or disappearance of time constants in this zone will give us information about the changes in the steel surface and the regeneration of the passive layer.

### 2.3. Electrochemical Chloride Extraction (ECE): Application and Effectiveness Monitoring

To detect the influence of the electro remediation treatment, only two of the three rebars (*S*_b_ and *S*_c_) were connected to the protection system, leaving the third rebar (*S*_a_) without connection to the current that results in ion migration.

**Figure 1:**
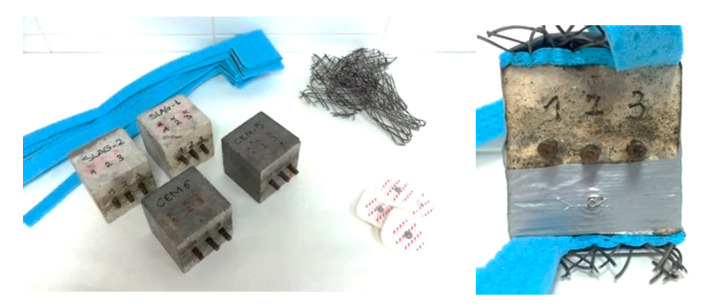
Specimens fabricated: steel 1 (*S*_a_), steel 2 (*S*_b_), and steel 3 (*S*_c_).

The titanium mesh (TiMMO), made by coating titanium with a combination electrically conductive metal oxide, was placed below the underside of prims, as an anode. The total area covered by the anode was 49 cm^2^. A wet sponge was placed between the specimen and the mesh, ensuring permanent electrolytic contact between the anode and cathode, as Figure 1 depicts. The Ag/AgCl textile repositionable electrode (3M™ Red Dot™) has been used to constantly monitor the electric potential of the different steels during the treatment.

The ECE treatment was applied potentiostatically. The voltage applied was increased in three steps during the tests, according to the total accumulated electric charge density reached, σ (calculated in each specimen according to the specimen area covered by the anode, 49 cm^2^). The procedure followed is explained in [33,52].

- The initial voltage applied was 12 V until the accumulated charge reached 1500 Ah/m^2^.
- Then, the voltage applied was increased to 16 V until the accumulated charge was 2000 Ah/m^2^.
- The maximum voltage applied was 20 V. The treatment was stopped when it reached 4500 Ah/m^2^.

This methodology was applied in all the cases except for the slag typology, as will be shown in the Results.

During the ECE treatment, once a week, the current source was disconnected, and electrical resistance, depolarized potential, and *R*_p_ after 24 h of depolarization were also taken on the three steel rebars. I_corr_ was calculated from *R*_p_ values using the Stern–Geary Equation (4).

After the ECE, the efficiency of the treatment was checked monitoring corrosion potential and corrosion rate and comparing these values before and after the treatment. Chloride profiles were also determined at the end of the treatment to know the chloride distribution and quantification. Total and free chloride contents were determined following the standard UNE 112010:2011 and the Rilem recommendations [53,54].

Other characterization techniques, such as MIP, pore-pressing technique to determine the pore solution composition, or FRX to determine profiles of different elements, which were used for the initial mortar characterization, were also used at the end of the tests.

## 3. Results and Discussion

### 3.1. Mortar Characterization

#### 3.1.1. Fresh Properties

The workability of concrete was measured in terms of slump, noted immediately after manufacturing the concrete. The slump test was carried out following EN 1015-3, determination of consistence of fresh mortar (by flow table), while the air content was measured according to EN 1015-7, using the pressure gauge method. In this way, density in fresh state was also calculated according to EN 1015-6. These results are reported in Table 2. It can be seen from this table that the final diameter after the slump test was within the target range of 225 to 230 mm. Even though no significant differences are detected concerning the workability, a reduction in the percentage of occluded air as well as densification is detected when using blended cements.

#### 3.1.2. Chemical Composition

The chemistry of SCMs is generally characterized by lower calcium content than Portland cement [35]. Thus, there are differences in the hydrates formed during hydration, which influence strength and durability.

The initial chemical composition of the mortars fabricated was determined by FRX at the age of 28 days. The results, presented as normalized metal oxides composition, are shown in Table 3. As expected, the blended mortars present higher content of silicates (main component of the pozzolanic additions) than the OPC mortars, decreasing the content of calcium and sodium.

#### 3.1.3. Mechanical Resistance

Compressive strength (4 determinations per mix) and flexural strength (2 determinations per mix) of the three mortar typologies at the age of 28 days are presented in Table 4. The higher strength is reached by the slag typology, being the CEM I mortar the one with the lowest value.

#### 3.1.4. Electrical Resistivity of the Mortars

Table 5 depicts the averaged electrical resistivity values at early age state (24 h) and after 28 and 100 days, which reflect the characteristic of the SCM because of their hydration capacity. At an early age, no big differences are detected concerning concrete resistivity, being the mortar containing nonblended OPC (CEM I) the one with higher resistivity and presenting the one containing CEM III (slag) the lowest value. Because of its longer hydration time, the slag samples show, after 28 days of curing, an averaged resistivity 27 times higher than the CEM I, presenting the other SCM tested (FA) having similar values to CEM I at those ages. These results are in concordance with the mechanical resistance values measured also at the same age (Table 4) and the total porosity also evaluated, which will be commented on later.

After curing time, specimens remained in wet conditions until the start of the ECE treatment. During this time (more than 100 days after fabrication), resistivity measurements were monitored, and the averaged values obtained after 100 days are also presented in Table 5. It is observed that the slag specimens reach resistivity values more than one order of magnitude higher than the rest. It demonstrates that the resistivity measurement can imply the different chemical and physical behaviors of different mineral admixtures in cementitious materials [55,56]. There could be two effects due to the incorporation of SCMs, physical densification of the microstructure, and pozzolanic reaction leading to the formation of secondary C-S-H. Both could lead to an increase in resistivity, which is more significant in the case of slag specimens than in FA.

### 3.2. Steel Electrochemical Characterization (before ECE)

#### 3.2.1. Corrosion Potential and Corrosion Rate Measured by LPR

**Figure 2:**
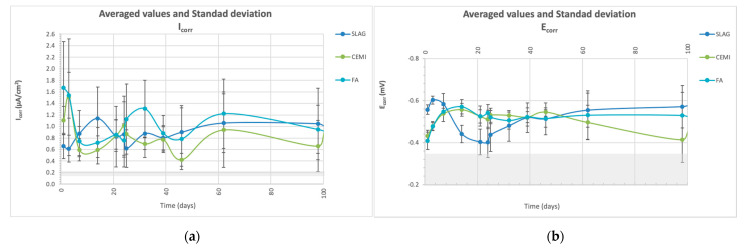
Electrochemical parameters (**a**) corrosion rate, I_corr_ and (**b**) corrosion potential, E_corr_ evolution. Averaged values and standard deviation. Threshold values that indicate the limit between passivation and corrosion are also indicated as a hatched area.

Corrosion characterization tests were carried out in the specimens with three rebars embedded. Figure 2 presents the corrosion potential and corrosion rate results for each mortar type (averaged of 6 determinations per mortar), obtained by the LPR technique, using the Stern-Geary formula [45]. Five percent chloride by mass of the cement added as NaCl to the mixing water provokes corrosion rates close to 1 μA/cm^2^ (high corrosion) in all cases. Corrosion potential values are in the range of −500 up to −580 mV (vs. Ag/AgCl). The mortar that presents lower corrosion rates is the OPC without additions (CEM I), presenting all the mortars containing SCMs’ higher corrosion ability.

It is well known that the SCMs provide better durability behavior in terms of corrosion, as the chloride diffusion through the concrete cover seems to be lower because of the pore structure refinement, in this case, especially, in slag specimens. Even though resistivity values and pore size distribution in slag specimens corroborate higher densification in these specimens and considering that pore pressing results confirm that slag mortar presents higher alkalinity than CEM I when chlorides are added during fabrication, no better behavior against corrosion is detected in the slag specimens. The higher consumption of portlandite in these specimens could be the reason for this behavior.

#### 3.2.2. Electrochemical Impedance Spectroscopy, EIS

**Figure 3:**
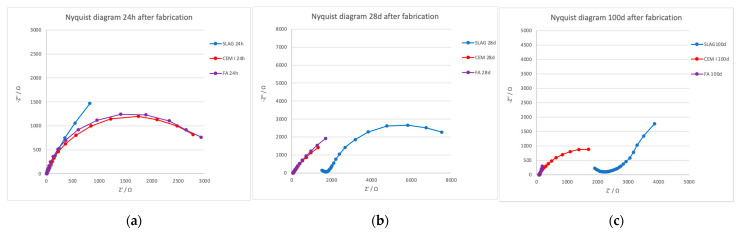
Nyquist diagrams obtained in the different mortars from early age up to 100 days after fabrication. Where Z’ is the real component and Z’’ is the imaginary component of the impedance vector. (**a**) 24h after fabrication; (**b**) 28 days after fabrication; (**c**) 100 days after fabrication.

This technique was used to study the mortar and the embedded rebar behavior from early age up to 100 days after fabrication. In Figure 3, representative spectra considering the central steels embedded in the mortars as a working electrode are presented (3 electrode measurements). All spectra were fitted to a Randles simple equivalent circuit [50], considering only one time constant in the process. The fitted results are presented in Table 6, where C is the capacitor, R_p_ the polarization resistance, R_s_ the electrical resistance, and CPE the constant phase element. As was observed in the resistivity measurements already presented, at an early age, the electrolyte resistance (R_s_) measured is similar in all specimens, increasing this value severely with curing time in the case of slag specimens, in which a second-time constant must be considered at high frequencies due to the mortar properties. A higher frequency range would be necessary to study these properties, implementing tests in a two-electrode arrangement instead of the three-electrode system used in this study.

Concerning *R*_p_ values studied at a low-frequency range (charge transfer resistance), an important increase is detected in FA and CEM I specimens from fabrication time up to 28 days after fabrication, with this value remaining almost constant in the slag specimen. That is, because of the higher alkalinity content, the steel presents higher R_p_ values in the initial time, which increases with the curing time because of the presence of chlorides that produce the steel depassivation. The results are in concordance with the LPR measurements presented before.

### 3.3. ECE Effects: Efficiency Indicators Evaluated during the Treatment

Results of the different parameters monitored during the ECE treatment in the four mortar typologies are presented.

#### 3.3.1. Surface Charged Density

Enough efficiency is usually assumed if a certain total electrical surface charge density σ (Ah/m^2^) has been passed [30]. However, because of the different microstructural characteristics of the different cement types and their different binding ability, this parameter is not reliable enough to inform on the reinstalling of passivity [57]. The total electrical charge density reached on CEM I and FA typologies was 4500 Ah/m^2^. That is, 2.6 times higher than the one reached in the slag typology (1740 Ah/m^2^), in which the recommended limit of 2000 Ah/m² was not reached.

**Figure 4:**
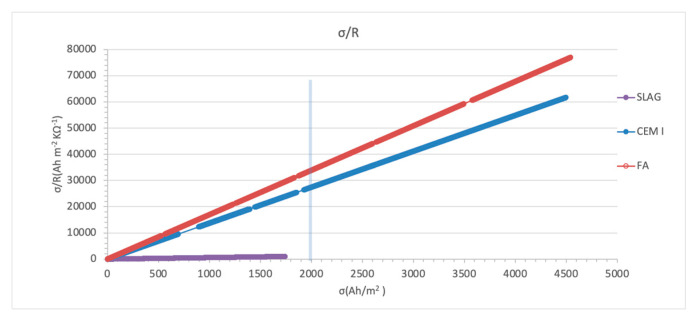
Charged density values normalized using the initial mortar resistivity (σ/*R*) represented vs. the accumulated charged density (σ).

These differences are due to the high electrical resistivity of this dosage, as shown in Figure 4. In this way, Figure 4 presents the accumulated charge density values normalized by the initial resistivity (σ/*R*), which is proposed by the authors as a more adequate indicator of the treatment progress.

#### 3.3.2. Depolarization Records during ECE

**Figure 5:**
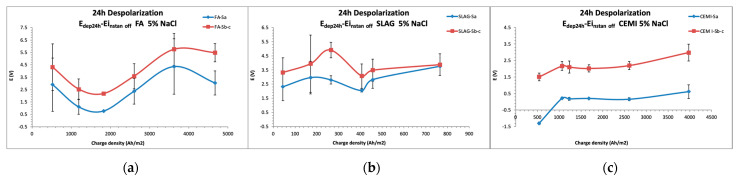
The 24 h depolarized potential averaged values registered during the treatment and standard deviation. (**a**) FA specimens; (**b**) SLAB specimens and (**c**) CEM I specimens.

The potential variations between the depolarized potential after 24 h of disconnection and the potential measured only 0.5 s after the disconnection (instant off potential) are presented in Figure 5.

An increase in depolarization with time is observed in general, except in the case of slag, due to the low efficiency in the treatment. A clear difference between the behavior of steels connected to treatment (*S*_b_ and *S*_c_) and not connected (*S*_a_) for tests performed on CEM I is observed. By contrast, in the case of FA, depolarizations reached by the nonconnected steel are similar to those of the connected steel, which is due to an increase in induced polarization in these cases, which could improve the protection of *S*_a_.

Maximum depolarization is detected in specimen CEM I when the accumulated charge density was near 4000 Ah/m^2^. In the case of FA, the maximum depolarization was reached under the 3000 Ah/m^2^ value.

Thus, it could be possible to deduce that the treatment efficiency is reached earlier in FA than in CEM I, obtaining a degree of protection even in the nonconnected steel in the case of FA.

#### 3.3.3. Electrochemical Impedance Spectroscopy

**Figure 6:**
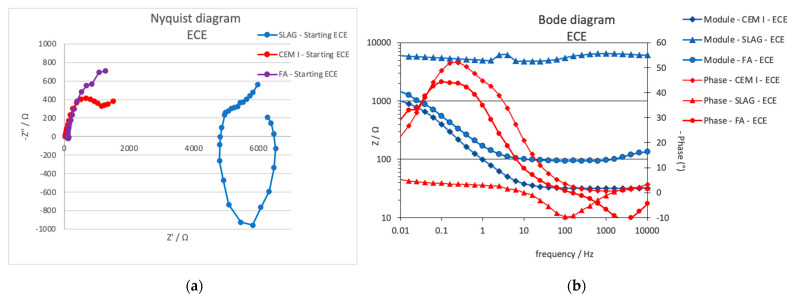
(**a**) Nyquist and (**b**) Bode diagrams obtained in the three mortar specimens in the first phase of the ECE treatment using the central rebar as working electrode, where Z is the impedance vector module and Z’ Z’’ the real and the imaginary components.

Electrochemical impedance spectroscopy measurements were taken during the ECE treatment. The measurements were carried out during the periodical depolarization times not only in the steels connected to the treatment, but also in the nonconnected one. In Figure 6, an example of the EIS typical representation, using Nyquist and Bode diagrams, is presented for the central steel connected to the treatment in the three specimen typologies studied (first stage of the treatment). The impedance module measured in the whole frequency range is more than one order of magnitude higher in the case of slag specimens. The high-frequency range shows also a capacitive behavior in this mortar typology, which is not detected in the other specimens.

**Figure 7:**
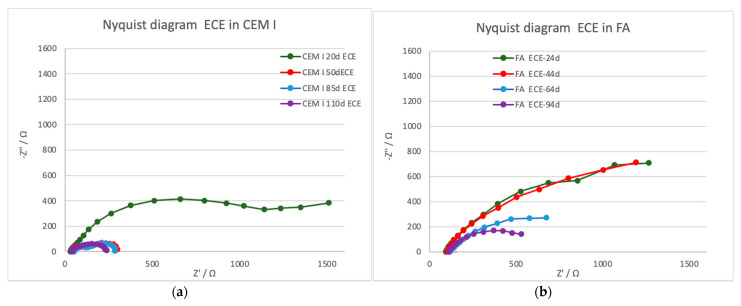
Nyquist diagrams showing the ECE treatment evolution for FA and CEM I mortar typologies. (**a**) CEM I specimens; (**b**) FA specimens.

Figure 7 presents some representative results obtained only in the specimens in which the ECE was effective (CEM I and FA specimens). Big interferences were detected due to the high resistivity and the residual polarization on the Slag specimens, so no clear semicircles appeared in the Nyquist diagram, and they have been discarded for interpretation.

**Figure 8:**
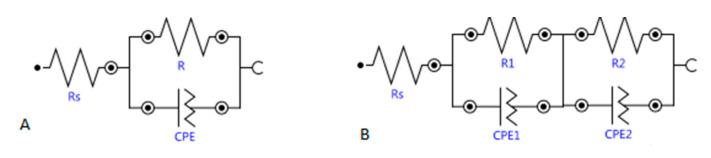
Equivalent circuits used for the EIS fitting. (**A**) Randles simple; (**B**) two serial RC circuits.

The treatment evolution registered by the EIS is different depending on the mortar typology. Two-time constants appear in the case of CEM I, whereas in the case of FA, only one-time constant is detected in the low-frequency range; thus, different faradaic processes are taking place in the different mortar–steel surface. Table 7 presents the characteristic parameters of the EIS spectra fitted to one- or two-time constant using equivalent circuits presented in Figure 8. While the *R*_s_ value (electrolyte resistance) does not vary significantly during the treatment in all of them, the *R* is due to the charge transfer, related to the double-layer capacity generated in the steel–mortar interface, presents different evolution in the different tests. A clear decrease (contraction of the semicircle) is detected in both CEM I and FA, which can be related to the good progress of the treatment.

**Figure 9:**
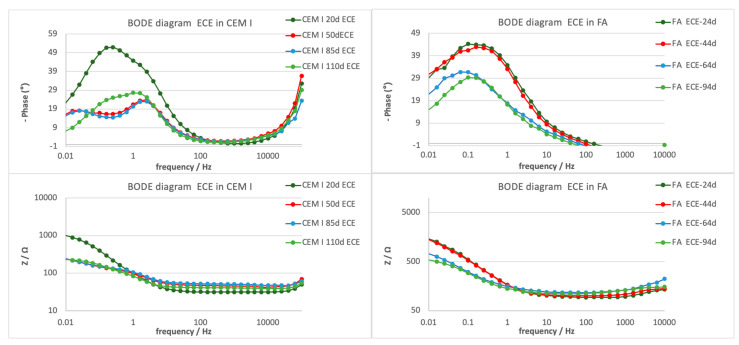
Bode diagrams showing the ECE treatment evolution in the different mortar typologies.

Looking at the phase angle evolution, a reduction of this parameter is clearly appreciated at the lower frequencies in both cases, CEM I and FA, as can be better appreciated in bode diagrams presented in Figure 9.

### 3.4. ECE Effects: Posttreatment Indicators

#### 3.4.1. Electrochemical Characterization: E_corr_ and I_corr_ (LPR and EIS Measurements)

**Figure 10:**
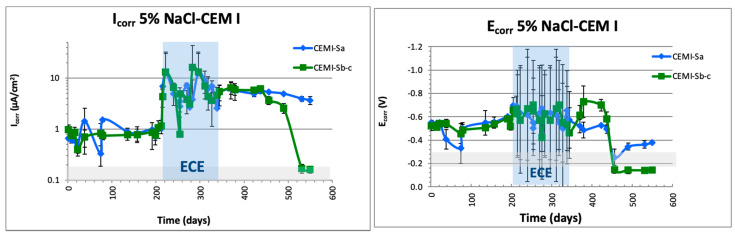

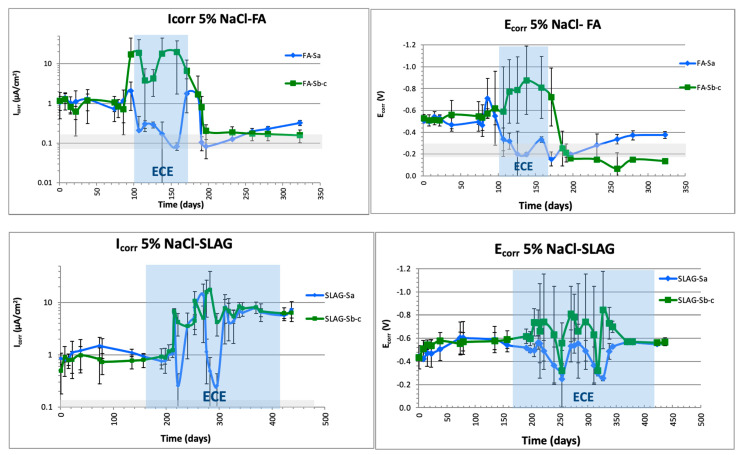
Corrosion potential and corrosion rate (E_corr_ and I_corr_) monitoring.

Figure 10 shows the monitoring of E_corr_ (vs. Ag/AgCl) and I_corr_ (by LPR method). The values during ECE were taken at the end of periodical 24-h depolarization.

About *E*_corr_ values, the tests performed on specimen CEM I show values before ECE treatment of high corrosion risk (−600 mV vs. Ag/AgCl electrode) in the three embedded steels, descending drastically (−180 mV vs. Ag/AgCl) a few days after the end of treatment to low corrosion risk values only in *S*_b_ and *S*_c_ (connected to ECE), while the nonconnected steel (*S*_a_) remains active (−400 mV vs. Ag/AgCl). The same behavior is obtained in FA specimens. E_corr_ values were more negative than −500 mV before treatment. After treatment, the nonconnected steel (*S*_a_) reached values close to the threshold given by the ASTM standard of high corrosion risk, −350 mV, which indicates uncertain corrosion risk in this case. On the other hand, steels connected to treatment reached values below the depassivation threshold set in the standard [58]. Slag specimens’ behavior differs a lot from the others. The higher electrical resistivity increases treatment time, but E_corr_ values at the end of ECE indicate the same high corrosion risk as before ECE.

About I_corr_ values measured by LPR, this parameter after completion of treatment and stabilization value (several weeks after disconnection) is considered to determine the quantitative reduction of corrosion in steel [59]. Figure 10 shows I_corr_ monitored before, during, and after the ECE treatment.

The CEM I samples present a decrease in I_corr_ from high to negligible values on the steels *S*_b_ and *S*_c_ (connected), whereas the *S*_a_ steel, not connected, does not reach passivation values after the treatment (>0.2 μA/cm^2^). This effect in the steel not connected to the treatment is in concordance with the first results obtained in [33]. In the case of FA, even though the tendency is similar to that in CEM I, the differences after treatment between steel *S*_a_ (not connected) and connected (*S*_b_ and *S*_c_) are smaller than that in the case of CEM I. In regard to SLAG typology, as shown in Figure 10, no re-passivation of the steels is detected.

**Figure 11:**
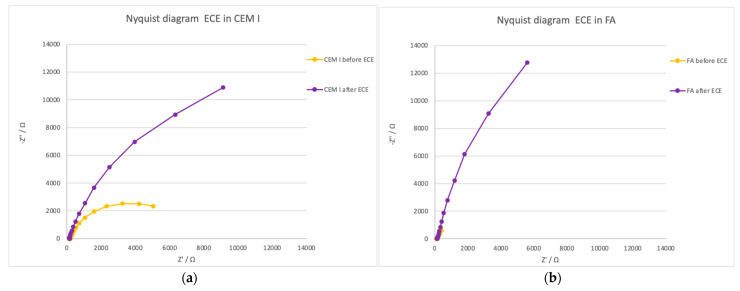
Nyquist diagrams obtained by the EIS in the central steel connected to the treatment. Measurements carried out just before treatment and after ECE. (**a**) CEM I specimens; (**b**) FA specimens.

Regarding the EIS study, Figure 11 shows the effect of the ECE in the central steel, connected to the treatment. An important increase in the charge transfer resistance is detected in the two cases in which the treatment was efficient. That means a drastic decrease in corrosion rate.

#### 3.4.2. Quantitative Chloride Profiles at the End of Treatment: Extraction Efficiency

When stable E_corr_ and I_corr_ values were reached after the end of the treatment, specimens were broken, and chloride content was determined. After the acid attack, quantitative determinations were made by the potentiometric method. Both free chlorides and total chlorides (not combined with cement phases and therefore soluble in water and liable to diffusion through the concrete pores) were determined following the recommended RILEM procedures [53,54].

**Figure 12:**
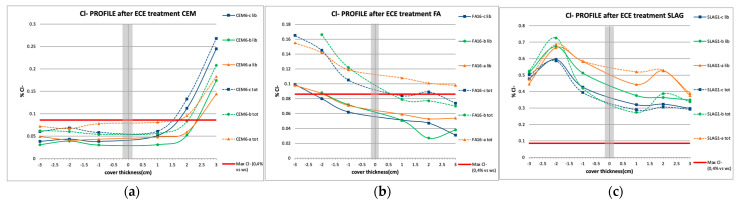
Chloride profiles determined in specimens after the ECE treatment. (**a**) CEM I specimens; (**b**) FA specimens; (**c**) SLAG specimens.

It can be outlined that FA and CEM I specimens have suffered a reduction of chlorides below the threshold of 0.4% Cl by weight of cement, established by different international structural concrete codes and in the bibliography as no corrosion risk (Figure 12). In general, in the area close to the steel not connected to the treatment, quite higher chloride concentrations are detected.

In this essay, the CEM I typology presents values below the limits set for both total and free chlorides in every rebar and at all depths, except in the area near the anode. An accumulation of chlorides is detected in this anodic area. In the case of FA, chloride accumulation in the area beyond the cathode is observed (Figure 12). That is, it was not possible to remove chlorides beyond the cathode efficiently. Note that the chloride reduction in the vicinity of the rebars is quite lower than those obtained in CEM I. A higher degree of chloride combination is detected on this mortar typology, being the difference between total and free chloride concentration higher than that in the case of CEM I. Nevertheless, the chemical combination of chloride ions is frequently considered as a beneficial effect in that it reduces the rate of chloride penetration in concrete, and its effect has recently been studied in ECE [42].

The analysis in the slag specimens shows that chloride content is over the limit established in all the cases. It is remarkable to mention that both free and total chloride contents determined close to the steel unconnected to the treatment are higher than the values obtained in the connected rebars, and a reduction in the chloride content is obtained. However, electrochemical results from Figure 10 show corrosion rates indicating active corrosion in all the steels, and in Figure 4, the charge density graph shows deficient progress of the treatment. So, the treatment is not effective on elements with this mineral admixture.

Trying to correlate the results provided by the non-destructive electrochemical technique evaluation after the treatment (quantification of the corrosion rate), with the presence of chloride-inducing corrosion, good concordance with the results shown in Figure 10 and Figure 11 has been obtained. Coincidences are clear for rebars connected to treatment, showing low I_corr_ values consistent with negligible levels of chloride ion concentration.

**Figure 13:**
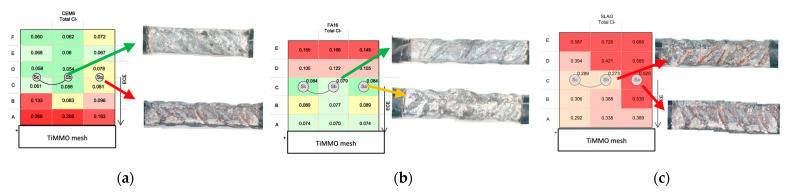
Visual inspection of the steels. (**a**) CEM I specimen; (**b**) FA specimen; (**c**) SLAG specimen.

Figure 13 represents the appearance of steels after the application of the ECE treatment. For each type, images of the steels connected and disconnected to the ECE treatment are shown. Differences in the connected and disconnected steels are clearly appreciated in CEM I, which are in concordance with the electrochemical parameters evaluated and the chloride amount found in the surroundings of these steels that concluded the passivation in the first one and the corrosion in the second. In the case of FA, the differences detected in both connected and unconnected steels were not so important, presenting the unconnected steel electrochemical corrosion values close to the passivation threshold. Note that the oxides formed in the unconnected steels are different in CEM y and FA. In the case of slag, where the treatment has not been effective, the formation of oxides is continuous along with both steels, demonstrating the inefficacy of this technique in this concrete application. Future analyses will be developed to study the oxides formed in each case.

### 3.5. ECE Additional Effects

#### 3.5.1. Pore Structure and Pore Solution

Cement hydration reaction results in two kinds of products: solid products and a pore system. A large number of the engineering properties of cement-based materials, such as strength, permeability and diffusivity, shrinkage and creep, and durability, are known to be intimately associated with characteristics of the pore system. The pore system in cement-based materials consists of four types of pores. These are as follows: (a) gel pores, which are micropores of characteristic dimension 0.5 to 10 nm. They constitute the internal porosity of the C-S-H gel phase; (b) capillary pores, which are mesopores with an average radius ranging from 5 to 5000 nm; the sizes of capillary and gel pores overlap, and the spectrum of pore sizes in a cement paste is continuous; (c) macropores due to deliberately entrained air; and (d) macropores due to inadequate compaction. The gel pores, which are mostly of 1.5 to 2.0 nm, do not influence the strength of concrete adversely through its porosity, although these pores are directly related to creep and shrinkage. Capillary pores and macropores, on the other hand, are responsible for the reduction in strength and elasticity.

The apparent porosity and pore size distribution of the mortars studied were determined at the age of 50 days and at the end of the treatment through MIP (mercury intrusion porosimetry).

**Figure 14:**
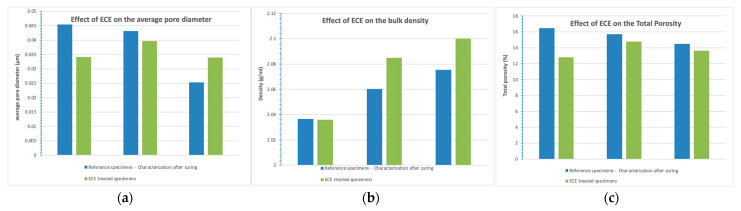
Influence of binder type in the pore structure before and after ECE treatment. (**a**) Average pore diameter; (**b**) bulk density; (**c**) total porosity.

As shown in Figure 14, considering the mortar fabricated with CEM I as a reference, initial porosity, and pore size were decreased when using slag (CEM III) and with the addition of FA to OPC. Fly ashes play an important role in the pore structure, partially filling the pores of the cement paste and reducing the total porosity, as has been also studied by other authors [43,60].

**Figure 15:**
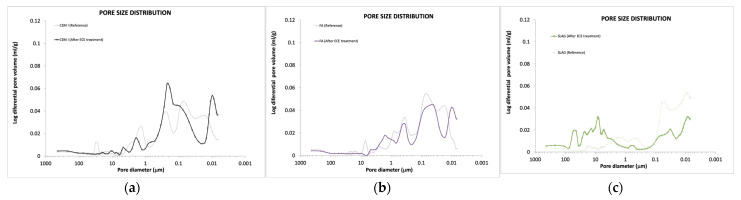
Influence of binder type in the pore size distribution before and after ECE treatment. (**a**) CEM I specimens (**b**) FA specimens; (**c**) SLAG specimens.

Despite this, pore size distribution obtained in CEM I and FA is quite similar, whereas, in the case of slag, there is an increase in the range of pores with a diameter lower than 50 nm (micropores and mesopores), decreasing the percentage of higher pores (mesopores and macropores) (Figure 15). That is, there is a pore refinement. As has been mentioned, a decrease in the number of macropores (diameter >5000 nm) leads to an increase in the mechanical resistance, as was detected in the initial mechanical tests.

The pore structure of the cement matrix may directly affect ionic transport under the treatment, being governed by the water-to-binder ratio, binder type, and aggregate portion in the mix. On the other hand, the pore diameter and the pore volume will be also affected by the treatment, because of the different anodic and cathodic reactions that take place in the cement matrix. Figure 14 shows the influence of binder type in the pore structure after ECE treatment. A decrease in total porosity is observed in all cases, which means in general bulk densification. The averaged pore diameter decreases in the case of CEM I and FA. A new separated maximum of a number of pores is detected in both cases at 0.009 μm, which means a pore refinement. On the other hand, an increase in the averaged pore diameter is detected in the case of slag. According to these results, it can be concluded that even though slag specimens also suffer densification, the averaged pore size increases, as there is an increase in the number of macropores.

The pore pressing technique allows extracting the pore solution from the mortar matrix to be analyzed. It provides important information about its alkalinity and the ability to passivate metal components embedded in these matrices. The pore solution is extracted under pressure, and pH is determined by a pH meter with a combination pH electrode and/or OH^−^ titration.

**Figure 16:**
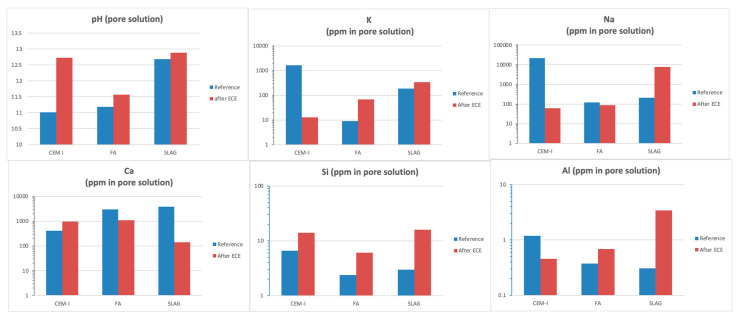
Influence of binder type in the pore solution before and after ECE treatment.

Figure 16 shows the recorded pH values of each of the specimens analyzed before and after the treatment application, as well as the pore solution composition measured by ICP.

In all cases, pH increases with the addition of SCM to the mix, varying initially from 11.02 when using OPC up to 12.68 with the use of slag. The pore solution composition also varies significantly, being the majorities’ elements in the pore solution when using CEM I, Na, and K, which are highly reduced when using SCMs. The increase in Ca in the pore solution when using SCMs is also remarkable, mainly with the addition of slag. In this way, it has been clearly demonstrated that higher pozzolanic activity is presented in slag mortar.

About the effect of ECE in the pore solution composition, it is remarkable that even though pH increases after treatment in all cases, OPC presents the most remarkable increase in alkalinity; that is, the hydroxyl generation during the cathodic reaction provokes the reaction with different cement phases considerably increasing the pH. In the case of blended cement, in which the initial alkalinity is higher than in OPC, the hydroxyl generation in the cathode surroundings also increases the pH, but in a more moderated way. The decrease in K, Na, and Al concentrations after treatment in the case of CEM I is also remarkable. On the other hand, an increase in the Si and Ca concentrations are detected in the same mortar typology, with the SCM behavior being the opposite. Thus, the effect of the treatment and the anodic and cathodic reactions provoke changes in the pore solution that differs from one mortar to another, improving its ability to passivate the steel.

#### 3.5.2. Bulk Chemical Composition

**Figure 17:**
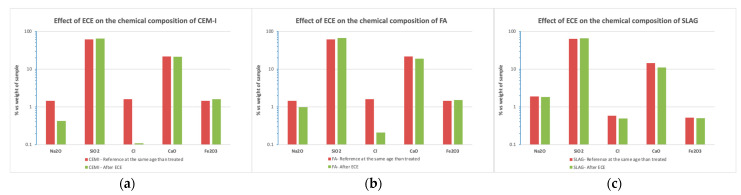
Influence of binder type in the bulk chemical composition before and after ECE treatment. (**a**) CEM I specimens; (**b**) FA specimens; (**c**) SLAG specimens.

The chemical composition was obtained using FRX analysis for CEM I, FA, and slag mortar powder samples. The results obtained from mortar specimens (before and after the application of the ECE treatment) are given in Figure 17. Note that the analysis is given in percentage of oxide composition, normalizing the results to 100%. The more important changes are detected, as expected, in the chloride reduction in CEM I and FA, as has been already commented. There is an important reduction in the proportion of sodium oxides, decreasing after the treatment in the case of CEM I and FA, as was also detected in the pore solution (Figure 16).

## 4. Conclusions

Two different SCMs with a cement replacement of 8% have been tested to determine the viability of ECE treatment on these conditions.

- Charge density normalized with resistivity values instead of charge density seems to be a better indicator to determine the end of the treatment, as the resistivity of the cementitious materials is one of the most important parameters to be considered to prior deduce if the material will have good behavior in an ECE treatment. Depolarized steel potential measured in periodical 24 h depolarizations also is shown as a good indicator of the treatment evolution. So, both parameters could be added to the ones already stablished to determine the end of the treatment.
- On the other hand, the resistance due to the charge transfer evaluated by the EIS during the treatment can provide important information about the treatment evolution. It is related to the double-layer capacity generated in the steel–mortar interface. The decrease (contraction of the semicircle) detected in both CEM I and FA can be related to the good progress of the treatment.
- Once the ECE has been stopped, corrosion rates determined by LPR or EIS provides reliable information about the steel passivation, and are in concordance with the destructive test applied (chloride profiles and visual inspection).
- All the parameters monitored indicate that in the case of FA, the passivation of the steels connected to treatment was reached in the same way as in plain CEM I specimens. A degree of protection is also detected in the nonconnected steel, which means that substitution of 8% in FA does not affect treatment efficiency and can also partially protect the metallic elements embedded in the same electrolyte but not connected to the treatment.
- On the other hand, in the case of using CEM III (slag), no steel passivation was reached.
- Notably, early age characterization and electrical resistivity values, which reflect the different characteristics of the mineral admixtures because of their hydration capacity, affect the ECE treatment. The slag mortar properties differ from the FA and CEM I and justify the low ionic mobility in this case.
- It is also noted that the different combination of chlorides (difference between total and free chlorides) affects the treatment efficiency. The addition of FA improves the combination of chloride with the cement phases; thus, total chloride remains high (as a result of the high degree combination), whereas free chloride content decreased with treatment. The chemical combination of chloride ions is frequently considered a beneficial effect in that it reduces the rate of chloride penetration in concrete.
- Changes in the pore structure and pore solution composition are detected. pH increases in all cases after treatment (more severely in the case of CEM I), whereas the ECE treatment reduces the porosity in all cases. Thus, the ECE treatment is able not only to reduce chloride but also to improve the mortar durability properties and its ability to passivate the steel.

## Figures and Tables

**Table 1:** Mortars dosage.

Components	OCP (CEM I)	Fly Ash (FA)	Blast-Furnace Slag
Cement *	675 g	621 g	675 g
Aggregate	2025 g	2025 g	2025 gr
Water	371 g	371 g	371 gr
Sodium chloride	56 g	55.70 g	56 gr
Fly ash (8% replacement)	—	54 g	—

* Type of cements: CEM I -42,5R/SR (for CEM I and FA specimens); CEM III/C 32,5N (for slag specimens).

**Table 2:** Early age characterization (fresh properties measured on each mortar mix).

Mortar	Density, kg/dm^3^	Occluded Air, %	∅ Initial, cm	∅ Final, cm
CEM I	2.05	9.0	12.5	22.5
FA	2.12	6.1	13.0	23.0
Slag	2.08	7.2	14.6	23.0

**Table 3:** Chemical composition of the mortars.

	SiO_2_	CaO	CO_2_	Al_2_O_3_	Na_2_O	Fe_2_O_3_	MgO	SO_3_	K_2_O	TiO_2_
CEM I	41.20	36.52	5.44	2.13	5.28	2.34	0.45	2.12	0.23	0.22
FA	68.22	18.08	5.30	1.88	1.33	1.69	0.28	1.29	0.18	0.13
Slag	67.91	14.41	4.62	3.25	1.92	0.65	2.37	2.20	0.27	0.18

**Table 4:** Mechanical resistance (EN 12504-1:2009).

Mortar	Flexural Strength			Compressive Strength		
	Average	St Dev	CoV(%)	Average	St Deb	CoV(%)
CEM I	7.29	1.01	13.92	35.69	3.47	9.71
FA	5.83	0.35	6.08	37.07	1.64	4.42
Slag	11.98	---	---	39.96	1.82	4.55

**Table 5:** Electrical resistivity at the age of 24 h, 28, and 100 days. Averaged values of six determinations.

	Age (days)		
ρ (KΩ·cm)	1	28	100
SLAG	0.093	32.584	38.255
SLAG (St Dev)	0.005	3.839	7.273
CEMI	0.192	1.380	1.497
CEMI (St Dev)	0.019	0.058	0.185
FA	0.161	1.177	1.838
FA (StDev)	0.010	0.088	0.107

**Table 6:** Characteristic parameters obtained from de fitting of the Nyquist diagrams to a Randle equivalent circuit. Only one specimen per mortar type was tested.

		*C* (*F*)	*R*_p_ (Ω)	*R*_s_ (Ω)	CPE
	Slag	0.00156	10,249	7.5925	0.99606
24 h	CEM I	0.00113	3343.2	9.0469	0.99645
	FA	0.00097	3388.1	7.9897	0.99682
	Slag	0.00075	7801.2	1769.7	0.99604
28 d	CEM I	0.00176	9079.2	70.728	0.99391
	FA	0.00133	12,082	63.868	0.99418
	Slag	0.00190	8392.6	2837.1	0.99675
100 d	CEM I	0.00511	3133.4	73.870	0.99396
	FA	0.00211	7564.9	98.702	0.99754

**Table 7:** Characteristic parameters obtained from the fitting of the Nyquist semicircle obtained by the EIS during the ECE treatment.

Mortar	Time (d)	CPE.Y0 (F)	*R*_p_.*R* (Ω)	*R*_s_.*R* (Ω)	CPE.N
CEM I	20	0.0051512	1309.000	29.291	0.9952800
	50	0.0017402	91.144	47.272	0.9977000
		0.0599460	199.010	119.820	0.9959300
	85	0.0016220	95.399	51.982	0.9977100
		0.0647850	168.700	130.620	0.9974500
	110	0.0032299	112.730	41.155	0.9973600
		0.0060538	208.480	40.247	0.9940000
FA	24	0.0067477	2358.700	90.951	0.9945700
	44	0.0062760	2536.000	86.788	0.9938500
	64	0.0120370	1019.700	113.900	0.9934700
	94	0.0083477	571.710	106.550	0.9945100

## Data Availability

The data presented in this study are available on request from the corresponding author. The data are not publicly available due to confidential research in progress.
